# Long intergenic non-coding RNA 00324 promotes gastric cancer cell proliferation via binding with HuR and stabilizing FAM83B expression

**DOI:** 10.1038/s41419-018-0758-8

**Published:** 2018-06-18

**Authors:** Zigui Zou, Tianshi Ma, Xuezhi He, Jinxing Zhou, Hongwei Ma, Min Xie, Yanhua Liu, Die Lu, Shihao Di, Zhihong Zhang

**Affiliations:** 1 Department of Pathology, The First Affiliated Hospital of Nanjing Medical University, Nanjing, China; 2 Department of Anatomy, Histology and Embryology, Research Centre for Bone and Stem Cells, Nanjing Medical University, Nanjing, China; 3 Department of Pathology, Zhenjiang First people’s hospital, Zhenjiang, China; 4 Central Laboratory, Suzhou Municipal Hospital, Nanjing Medical University Affiliated Suzhou Hospital, Suzhou, China; 5 Department of Oncology, Affiliated Xuzhou Central Hospital, Xuzhou Medical College, Xuzhou, China

## Abstract

Substantial evidence shows that long non-coding RNAs (lncRNAs) participate in many biological mechanisms, and their dysregulation are also involved in the development and progression of cancers, including gastric cancer (GC). Long intergenic non-coding RNA 00324 (LINC00324), a 2115 bp ncRNA, is located on chromosome 17p13.1. The biological function and molecular mechanisms of LINC00324 in GC remains undiscovered. In this paper, we found that the expression level of LINC00324 was significantly upregulated in GC tissues compared with the corresponding normal tissues. The overexpression of LINC00324 was correlated with advanced TNM stage, larger tumor size, and lymph node metastasis as well as poor prognosis. Further experiments revealed that knockdown of LINC00324 could suppress the proliferation of GC cells. RNA transcriptome sequencing technology revealed that FAM83B may be a significant downstream target gene of LINC00324. LINC00324 could combine with the RNA-binding protein (RBP) human antigen R (HuR) and thus stabilize the expression of FAM83B. Moreover, rescue assays showed that the reduced FAM83B expression partially reversed the promotion of cell growth in GC induced by the overexpression of LINC00324. In conclusion, our study revealed that LINC00324 acted as an oncogene in tumorigenesis and progression, suggesting that it could be a new biomarker in diagnosis and prognosis of GC.

## Introduction

Gastric cancer (GC) is a kind of common malignancy of the digestive system. The incidence of GC ranked fifth worldwide, and it is becoming the second leading cause of cancer death with 984,000 incident cases and 841,000 deaths occurring globally in 2013^1^. Although there is a steady decline in GC incidence and mortality rates, most GC patients are diagnosed at advanced stages. It means they missed the optimal opportunity for radical gastrectomy, which is still the only way to cure gastric cancer currently^2–4^. Thus, a clearer understanding of the pathogenesis and molecular mechanisms of GC is urgently needed to help us find more effective biomarkers and targets for GC diagnosis and therapy^5^.

The human genome sequencing project brings it to light that only 2% of the human genome encodes proteins, while the rest of RNAs without protein-coding capacity are known as non-coding RNAs (ncRNAs)^6, 7^. Generally, ncRNAs are divided into long ncRNAs (lncRNAs) (>200 nt) and small ncRNAs (≤200 nt)^8^. Although ncRNAs used to be considered as transcriptional “noise”, people currently realize the vital role of ncRNAs and pay more attention to them, especially to lncRNAs^9^. LncRNAs participate in many biological mechanisms, such as cell proliferation, apoptosis, migration, signaling, and differentiation^10–12^. LncRNAs can regulate gene expression at transcriptional and post-transcriptional levels, moreover, they are involved in the pathogenesis of various diseases, including cancers^13–15^. For instance, MALAT-1 can bind to the RNA-binding protein HuR, which negatively regulated CD133 and suppressed epithelial-to-mesenchymal (EMT) transition in breast cancer^16^. LINC01234 was significantly overexpressed in GC and functioned as a ceRNA for miR-204-5p, leading to the derepression of its endogenous target core-binding factor β (CBFB)^17^. The pseudogene DUXAP8 was upregulated in non-small-cell Lung Cancer (NSCLC), and it can bind to EZH2 and LSD1 to repress the transcription of EGR1 and RHOB epigenetically, which was involved in the cell proliferation and invasion of NSCLC^18^. Therefore, there is no doubt that lncRNAs are key factors in tumorigenesis and development, but the overall pathophysiological mechanisms of lncRNAs on GC remain to be determined.

HuR, an RBP, plays a significant role in mediating post-transcriptional regulation in various cancers^19, 20^. In addition, HuR can enhance the stability of mRNA and thus induce lncRNAs expression by binding to Adenylate-Urydinilate rich elements (AREs) in 3′-untranslated region^21, 22^. Although HuR is mainly located in the nucleus, its translocation from the nucleus to the cytoplasm has been involved in tumor development^21, 23^. HuR has been identified to combine with MALAT1, and the complex can repress CD133 Expression and Suppress EMT in Breast Cancer^16^. It has also been reported that HuR promotes the progression of bladder cancer by competitively binding to HOTAIR with miR-1^24^. Moreover, lncRNA UFC1 can bind to HuR, thereby enhancing the stability of β-catenin, its target mRNA, and promote the progression of liver cancer^25^.

The family with sequence similarity 83 member B (FAM83B) has been identified as an oncogene that can promote the transformation of immortalized human mammary epithelial cells (HMECs) by the validation-based insertional mutagenesis (VBIM) strategy and it is a key intermediary in EGFR/RAS/MAPK signaling^26^. FAM83B has also been reported to activate PI3K/AKT/mTOR signaling pathway, thereby promoting cell proliferation, anchorage-independent growth (AIG) and tumorigenicity in breast cancer^27^. Moreover, FAM83B was significantly upregulated in pancreatic ductal adenocarcinoma (PDAC) and lung squamous cell carcinoma (SCC) and was related to poor prognosis^28, 29^.

Long intergenic non-protein-coding RNA 324 (LINC00324), a 2115 bp ncRNA, is located on chromosome 17p13.1. The biological functions of LINC00324 in GC had not been explored. In this study, we found that LINC00324 was significantly upregulated in GC tissues compared with the corresponding adjacent normal tissues and the upregulation of LINC00324 was also associated with advanced TNM stage, larger tumor size, lymphatic metastasis, and poor prognosis of GC patients. Furthermore, our research indicated that LINC00324 can enhance the stability of FAM83B through binding to HuR, thereby promoting cell proliferation in GC.

## Results

### LINC00324 expression was upregulated in human GC tissues and cell lines

**Fig. 1:**
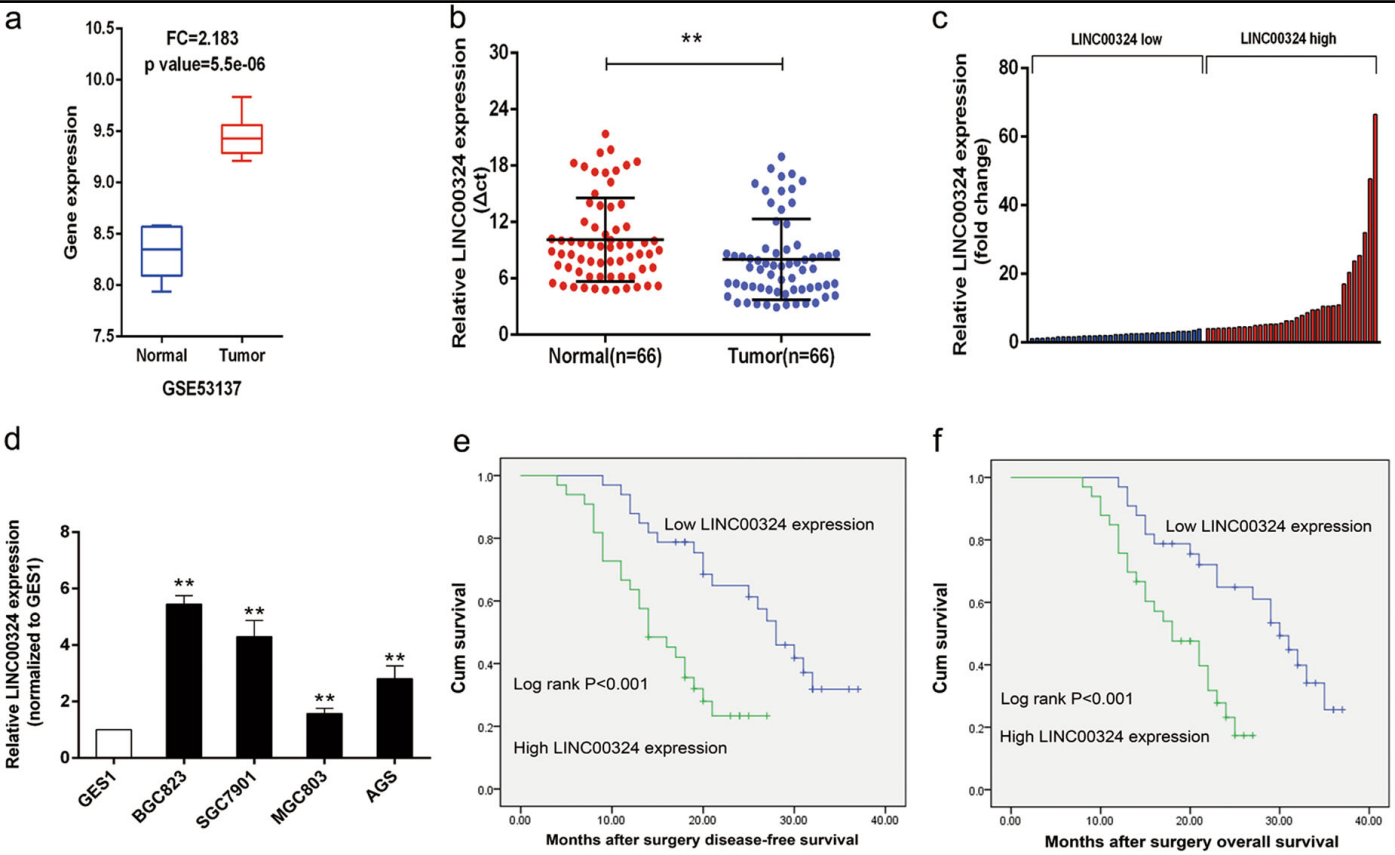
Relative expression of LINC00324 in GC tissues and cell lines as well as its clinical significance. **a** Relative LINC00324 expression in human gastric cancerous tissues (*n* = 6) compared with noncancerous tissue (*n* = 6) via GSE53137 data analysis (**b**) Relative expression of LINC00324 in GC tissues (*n* = 66) compared with their corresponding adjacent non-tumor tissues (*n* = 66) were analyzed by qRT-PCR and normalized against GAPDH expression. The data are presented as the delta CT value. **c** The GC patients were divided into two groups according to the expression of LINC00324. **d** qRT-PCR analysis of LINC00324 expression in normal gastric epithelium cell line (GES1) and GC cells. **e**, **f** Kaplan–Meier disease-free survival and overall survival curves according to LINC00324 expression levels. Error bars indicate mean ± standard errors of the mean. *P < 0.05, **P < 0.01

In this study, we first analyzed the expression levels of LINC00324 in human GC tissues by using raw microarray data downloaded from GEO (GSE53137)^30^, and found that LINC00324 expression levels were upregulated in GC tissues compared with the corresponding adjacent nontumorous tissues (Fig. 1a).Then, the expression levels of LINC00324 were investigated in 66 paired GC tissues and adjacent normal tissues by qRT-PCR and normalized to GAPDH. Figure 1b showed that LINC00324 expression was significantly upregulated in GC tissues compared with the corresponding adjacent normal tissues. In order to evaluate the relationship between LINC00324 expression and clinicopathological features in patients suffering from GC, 66 GC patients were divided into two groups: a relatively high LINC00324 expression group (*n* = 33, LINC00324 expression ratio ≥ median) and a relatively low LINC00324 expression group (*n* = 33, LINC00324 expression ratio < median) (Fig. 1c).The clinicopathological features of 66 gastric carcinoma patients were shown in Table 1.It was obvious that the relatively high LINC00324 expression group was more correlated with advanced TNM stage (*p* = 0.012), larger tumor size (*p* = 0.004) and lymph node metastasis (*p* = 0.020) than the relatively low LINC00324 expression group. However, the expression level of LINC00324 was irrelevant to other parameters such as gender (*p* = 0.804), age (*p* = 0.218) in GC. We also detected the expression level of LINC00324 in human GC cell lines using qRT-PCR. As shown in Fig. 1d, the LINC00324 expression was upregulated in GC cell lines and showed a much higher level in SGC7901 and BGC823 cells than other GC cell lines.

**Table 1:** Relationship between LINC00324 expression and clinicopathological characteristics of GC patients

Characteristics	*N*(%)	LINC00324		*P*
		High No. cases (33)	Low No. cases (33)	Chi-squared test *P*-value
Gender				0.804
Male	29(43.9%)	14	15	
Female	37(56.1%)	19	18	
Age				0.218
≤65	31(47.0%)	13	18	
>65	35(53.0%)	20	15	
TNM Stage				0.012*
I + II	26(39.4%)	8	18	
III	40(60.6%)	25	15	
Lymph node metastasis				0.020*
Negative	23(34.8%)	7	16	
Positive	43(65.2%)	26	17	
Tumor size				0.004*
≤5 cm	21(31.8%)	5	16	
>5 cm	45(68.2%)	28	17	
HP infection				0.211
Negative	27(40.9%)	11	16	
Positive	39(59.1%)	22	17	

^*^P < 0.05 was considered significant

### Association between LINC00324 expression and patient survival

The Kaplan–Meier analysis and log-rank test were used to evaluate the relationship between the expression level of LINC00324 and the prognosis of GC patients after gastrectomy and disease-free survival (DFS) and overall survival (OS) curves were plotted according to LINC00324 expression level (Fig. 1e, f). We can obviously find that GC patients with high LINC00324 expression had poorer DFS (*P* < 0.001) and OS (*P* < 0.001) than those with low LINC00324 expression. Multivariate analysis including gender, age, TNM stage, Lymph node metastasis, tumor size, HP infection, and LINC0324 expression revealed that TNM stage (*P* < 0.001) and LINC00324 expression (*P* < 0.001) were independent prognostic factors of DFS and OS (Table 2). Put these results together, LINC00324 can probably be a useful marker for the prognosis or progression of GC.

**Table 2:** Multivariate analysis of prognostic factors of survival

Parameters	Disease-free survival (DFS)		Overall survival (OS)	
	HR (95% CI)	*P*-value	HR (95% CI)	*P*-value
Gender	0.334–1.180	0.149	0.338–1.209	0.169
Age	0.615–2.230	0.630	0.578–2.156	0.742
TNM stage	0.021–0.163	0.000*	0.024–0.177	0.000*
Lymph node metastasis	0.295–1.298	0.204	0.247–1.167	0.116
Tumor size	0.402–2.301	0.931	0.406–2.541	0.972
HP infection	0.599–2.444	0.594	0.566–2.313	0.709
LINC00324 expression	0.021–0.192	0.000*	0.025–0.225	0.000*

*HR* Hazard ratio, *CI* Confidence interval

*P < 0.05 was considered significant

### LINC00324 promotes GC cell proliferation in vitro

**Fig. 2:**
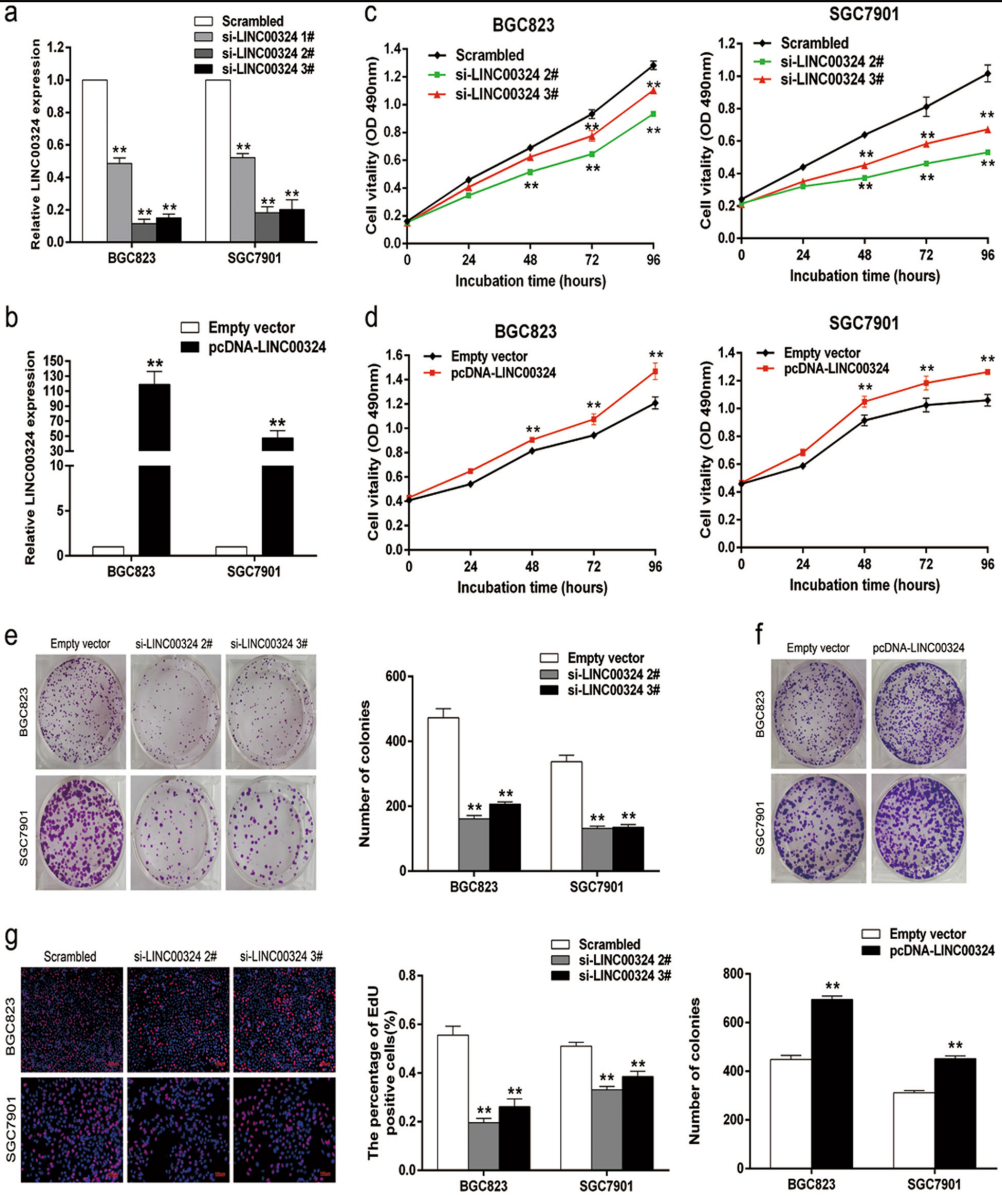
LINC00324 promotes GC cell proliferation in vitro. **a** qRT-PCR analysis of LINC00324 expression in BGC823 and SGC7901 cells transfected with control (scrambled), si-LINC00324 1#, si-LINC00324 2#, and si-LINC00324 3#. **b** qRT-PCR analysis of LINC00324 expression in BGC823 and SGC7901 cells transfected with empty vector and pcDNA-LINC00324. **c**, **d** MTT assays were performed to determine the viability of BGC823 and SGC7901 cells treated with si-LINC00324 or pcDNA-LINC00324. **e** Colony-formation assays were performed to detect the proliferation of si-LINC00324-transfected BGC823 and SGC7901 cells. Colonies were counted and captured. **f** Up: Colony-formation assays were performed to detect the proliferation of pcDNA-LINC00324-transfected BGC823 and SGC7901 cells. Down: Colonies were counted and captured. **g** EdU staining assays were used to determine the proliferation of si-LINC00324 transfected BGC823 and SGC7901 cells. EdU-positive cells were counted and captured. Values are shown as the mean ± standard errors of the mean based on three independent experiments. *P < 0.05, **P < 0.01

To investigate the function role of LINC00324 in GC, we designed three different LINC00324 siRNAs and then transfected them into SGC7901 and BGC823 cells. The qRT-PCR analysis was performed 48 h after transfection to detect whether all of the LINC00324 siRNAs were transfected into cells effectively. The results showed that si-LINC00324 2# and 3# had higher interference efficiency than si-LINC00324 1# in both SGC7901 and BGC823 cell lines (Fig. 2a). Therefore, we chose si-LINC00324 2# and 3# for the later experiments. At the same time, we transfected GC cell lines with pcDNA 3.1-LINC00324 expression vector to induce the ectopic overexpression of LINC00324. Similarly, the qRT-PCR analysis was used to assess the expression of LINC00324 and we found that its expression level was significantly upregulated in the GC cells which were transfected with pcDNA 3.1-LINC00324 compared with those transfected with the empty vector (Fig. 2b).

MTT assays showed that the proliferation of SGC7901 and BGC823 cells was significantly inhibited after knockdown of LINC00324 expression compared with the respective controls (Fig. 2c). In contrast, overexpression of LINC00324 promoted cell proliferation in both SGC7901 and BGC823 cells (Fig. 2d). The colony-formation assays showed the similar results. The clonogenic survival was significantly decreased when LINC00324 expression was downregulated, but increased when it was upregulated (Fig. 2e, f). Ethynyl deoxyuridine (EdU) (red)/DAPI (blue) immunostaining also verify the result (Fig. 2g & Supplementary Figure S1a). These data suggested that LINC00324 may act as an oncogene to promote the proliferation of GC cells.

### LINC00324 promotes GC cell migration and invasion in vitro

**Fig. 3:**
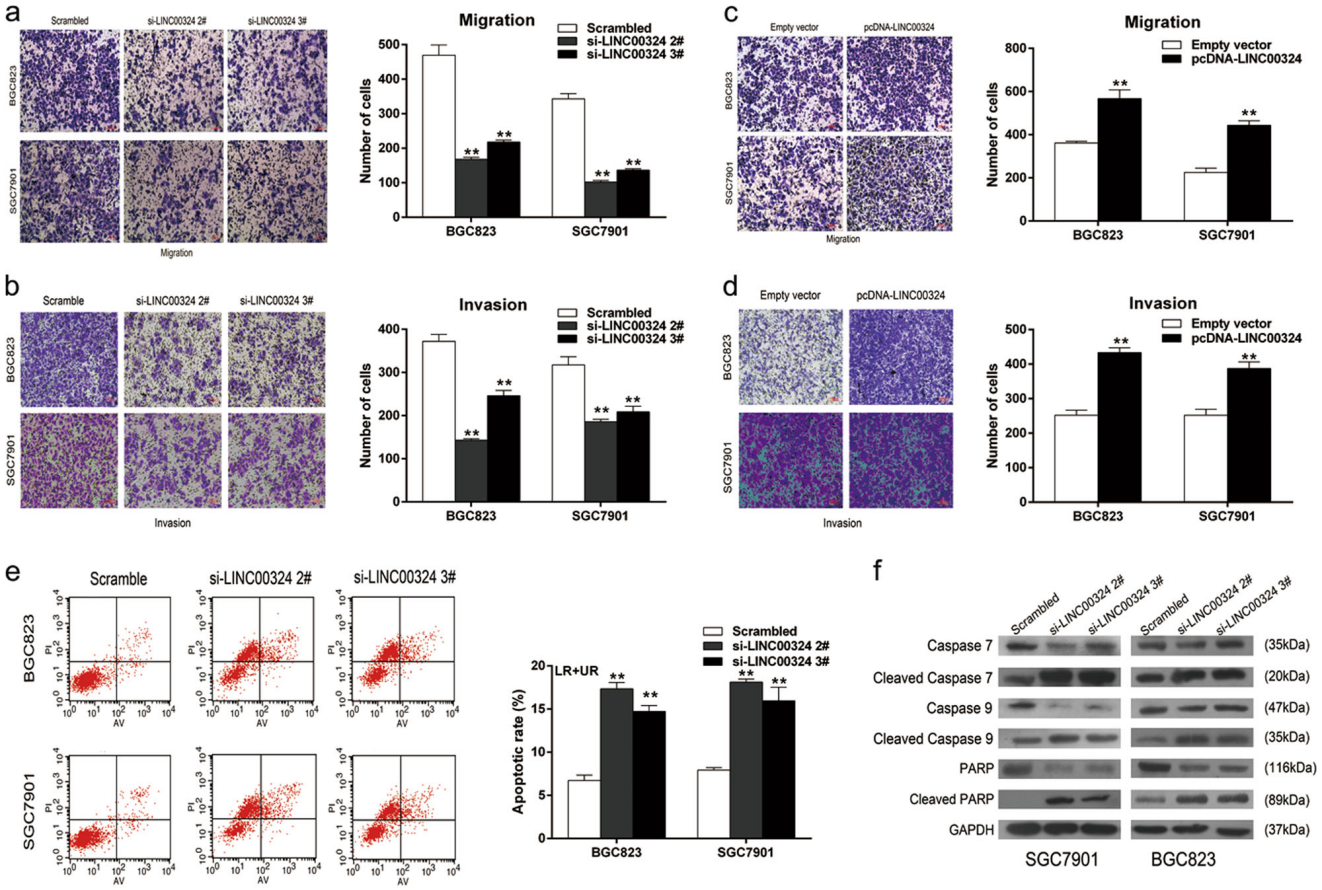
Effects of LINC00324 on the apoptosis, migration, and invasion of GC cells in vitro. **a**, **b** Transwell assays were performed to detect changes in migratory and invasion abilities of BGC823 and SGC7901 cells transfected with si-LINC00324 2#, 3# or scrambled. **c**, **d** Migration and invasion ability was investigated by transwell assays in BGC823 and SGC7901 cells transfected with pcDNA-LINC00324 or empty vector. **e** Apoptotic rates of cells were analyzed by flow cytometry. LR: early apoptotic cells, UR: terminal apoptotic cells. **f** Western blot analysis of apoptosis-related proteins in SGC7901 and BGC823 cells transfected with si-LINC00324 2#, 3#, or scrambled. GAPDH protein was used as an internal control. Values are shown as the mean ± standard errors of the mean based on three independent experiments. *P < 0.05, **P < 0.01

To investigate whether the expression level of LINC00324 was related to the migration and invasion of GC cells, we performed the transwell assays. The number of GC cells passing through the basement membrane was obviously decreased after downregulating the LINC00324 expression (Fig. 3a, b). In contrast, overexpression of LINC00324 promoted the migratory and invasion ability of GC cells (Fig. 3c, d). These data suggest that LINC00324 can promote the migratory and invasion ability of SGC7901 and BGC823 cells.

### Downregulation of LINC00324 induces apoptosis of GC cells

The level of apoptosis was one of factors which contributed to cell growth. To further detect whether the LINC00324 expression was relevant to the apoptosis level in GC cells, we performed flow-cytometric analysis. The results showed that the proportion of apoptotic cells treated with LINC00324 siRNAs were significantly increased (Fig. 3e). The level of cell cycle regulation was another factor contributed to cell growth. However, there were no significant differences in the proportion of cells at different stages of cell cycle (data not shown). In addition, western blot analysis was also performed to verify the previous results. The results revealed that the protein levels of cleaved caspase-7, cleaved caspase-9, and cleaved PARP were increased in GC cells treated with LINC00324 siRNAs (Fig. 3f). These data suggested that LINC00324 may inhibit GC cell apoptosis and be associated with GC progression.

### LINC00324 promotes tumorigenesis of GC cells in vivo

**Fig. 4:**
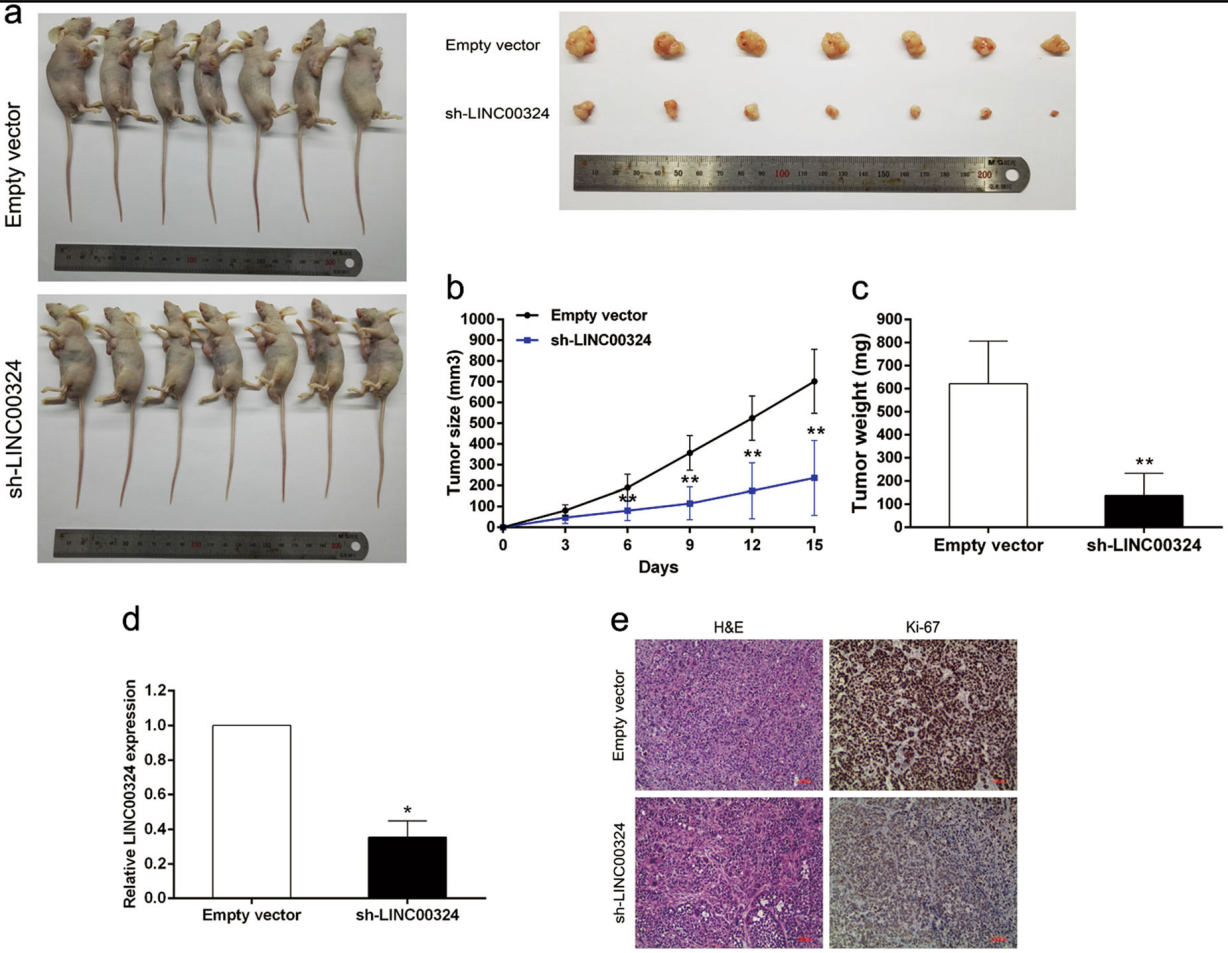
Effects of LINC00324 on GC tumorigenesis in vivo. **a** Empty vector or sh-LINC00324 was transfected into SGC7901 cells, which were then injected into the BALB/c-nude mice (*n* = 7), respectively. **b** Tumor volumes were calculated after injection every three days. Points, mean (*n* = 7); bars indicate SD. **c** Tumor weights were measured after tumor removal. **d** qRT-PCR was performed to detect the average expression of LINC00324 in xenograft tumors (*n* = 7). **e** The tumor sections were under H&E staining and IHC staining using antibodies against ki-67. *P < 0.05, **P < 0.01

To explore whether the expression level of LINC00324 could influence tumorigenesis in vivo, SGC7901 cells transfected with sh-LINC00324 or empty vector were inoculated into nude mice. All mice developed xenograft tumors at the injection site. Fifteen days after injection, we noticed that the tumors were much smaller in the sh-LINC00324 group than in the empty vector group (Fig. 4a). Moreover, tumor growth was significantly slower in the sh-LINC00324 group compared with that in the empty vector group (Fig. 4b). In addition, the average tumor weight was obviously lower in the sh-LINC00324 group than that in the empty vector group (Fig. 4c). QRT-PCR analysis was then used to analyze the expression level of LINC00324 in the xenograft tumor tissues. The results revealed that the expression level of LINC00324 was much lower in tumor tissues of the sh-LINC00324 group compared with the empty vector group (Fig. 4d). Immunohistochemical (IHC) anlaysis was used to analyze Ki67 levels in tumor tissues. The tumors developed from SGC7901 cells transfected with sh-LINC00324 showed lower density of Ki67 than tumors formed form cells transfected with empty vector (Fig. 4e). These data indicated that overexpression of LINC00324 was relevant to the tumorigenesis and proliferation capacity of GC cells in vivo.

### LINC00324 can bind with HuR

Based on the above study, we found that LINC00324 was involved in the progress of GC and we decided to further investigate its possible regulatory mechanism in GC.

**Fig. 5:**
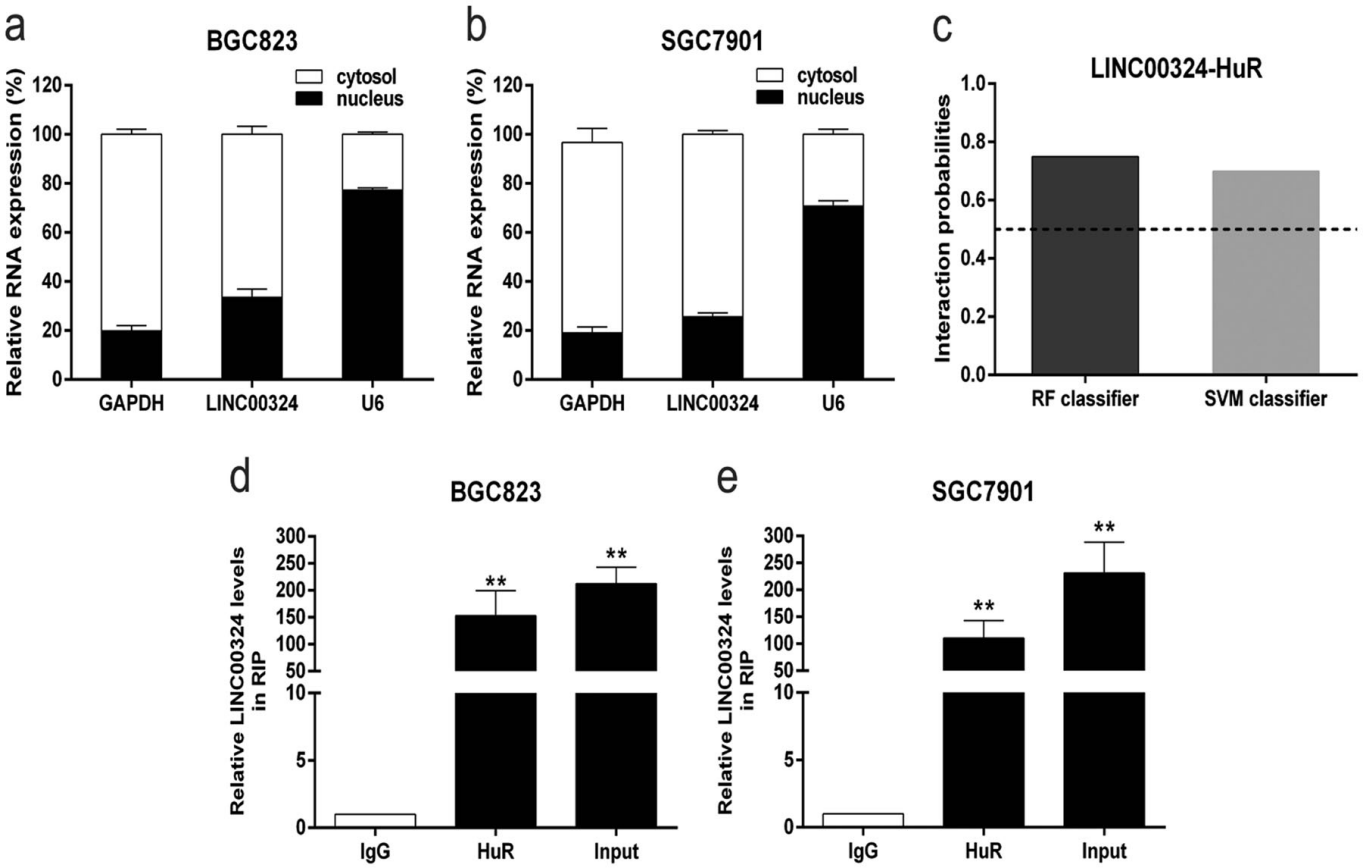
LINC00324 can bind with HuR. **a**, **b** The expression levels of LINC00324 in the cytoplasm or nucleus of BGC823 and SGC7901 cells were detected by qRT-PCR. GAPDH was used as a cytosol marker and U6 was used as a nuclear marker. **c** The prediction of the interaction probabilities of LINC00324 with RNA-binding protein HuR by Bioinformatics (http://pridb.gdcb.iastate.edu/RPISeq/). Predictions with probabilities >0.5 were considered “positive”, indicating that the corresponding RNA and protein are likely to interact. **d**, **e** RNA immunoprecipitation experiments were performed in BGC823 and SGC7901 cells and the coprecipitated RNA was subjected to qRT-PCR for LINC00324. The fold enrichment of LINC00324 in HuR RIP is relative to its matching IgG control. Values are shown as the mean ± standard errors of the mean based on three independent experiments. *P < 0.05, **P < 0.01

As shown in Fig. 5a, b, LINC00324 expression was higher in the cytosol than the nucleus in both BGC823 and SGC7901 cell lines, which suggested that LINC00324 may play a major regulatory role at post-transcriptional level. HUR, an RBP, can enhance the stability of mRNA and thus induce the expression of lncRNA^21, 22^. On the basis of these data, we then used Bioinformatics (http://pridb.gdcb.iastate.edu/RPISeq/) to predict whether LINC00324 can bind to HuR. The scores for RF Classifier and SVM Classifier are, respectively, 0.75 and 0.7 (Fig. 5c), indicating that LINC00324 stands a good chance of binding to HuR. Given this background, RNA immunoprecipitation (RIP) was performed to verify our surmise. The RIP assay revealed that LINC00324 highly bound to HuR in both SGC7901 and BGC823 cells (Fig. 5d, e).

### LINC0324 enhances the stability of FAM83B through binding to HuR

**Fig. 6:**
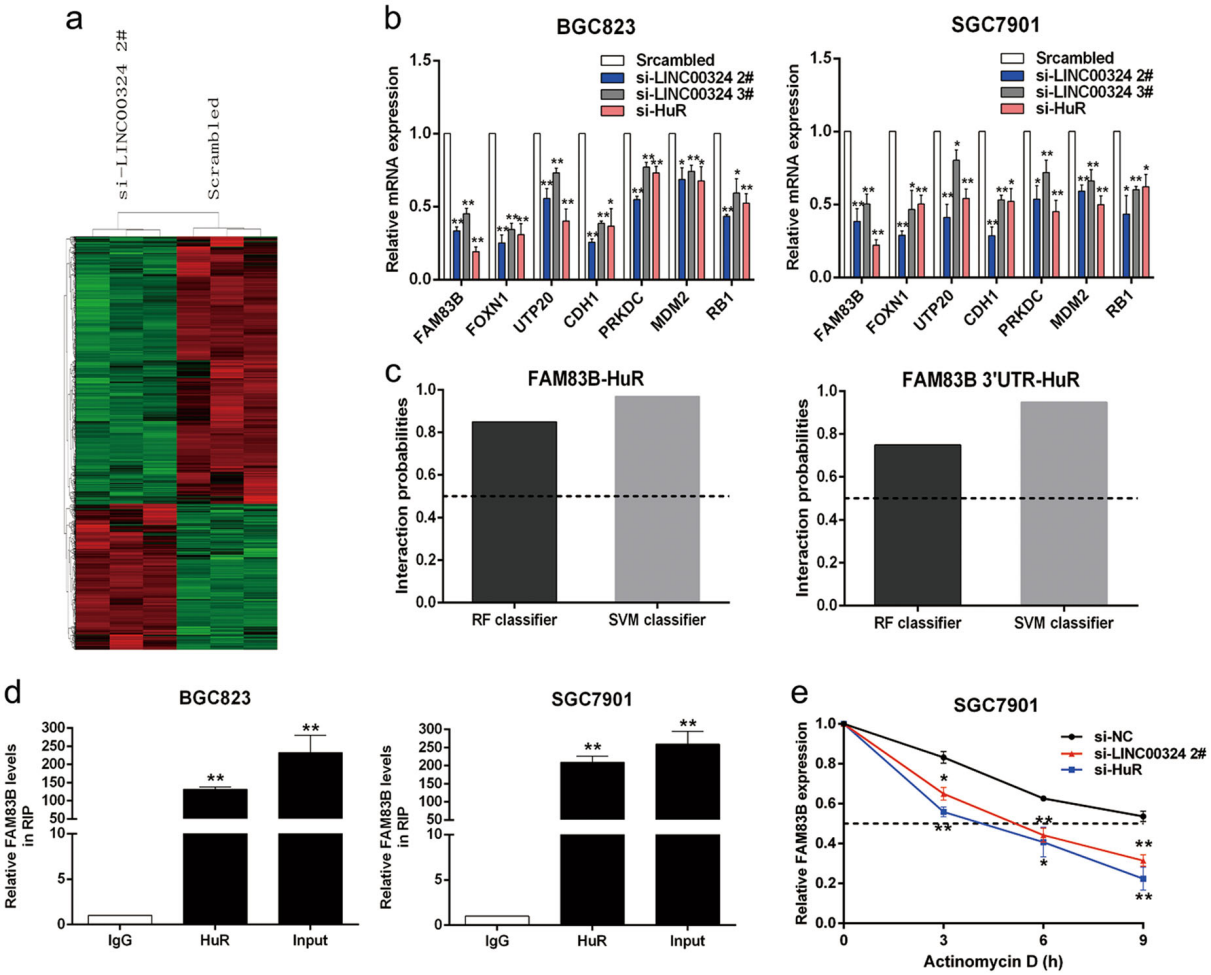
LINC0324 enhances the stability of FAM83B through binding to HuR. **a** Mean centered, hierarchical clustering of transcripts altered in GC cells treated with scrambled siRNA or si-LINC00324 2#, with three repeats. **b** qRT-PCR analysis was used to validate the changes of several mRNAs involved in cell proliferation and cell migration upon LINC00324 or HuR depletion. **c** The prediction of the binding abundance between HuR and FAM83B by Bioinformatics (http://pridb.gdcb.iastate.edu/RPISeq/). Predictions with probabilities > 0.5 were considered “positive”, indicating that the corresponding RNA and protein are likely to interact. **d** RNA immunoprecipitation experiments were performed in BGC823 and SGC7901 cells and the coprecipitated RNA was subjected to qRT-PCR for LINC00324. The fold enrichment of FAM83B in HuR RIP is relative to its matching IgG control. **e** RNA stability assays were performed using Actinomycin D to disrupt RNA synthesis in SGC7901 cells, and the degradation rates of the FAM83B mRNAs were measured every 3 h. Values are shown as the mean ± standard errors of the mean from three independent experiments. *P < 0.05, **P < 0.01

In order to obtain the target genes regulated by LINC00324, we performed RNA transcriptome sequencing to assess the gene expression profiles of SGC7901 cells in which the expression level of LINC00324 was suppressed.SGC7901 cells were transfected with a scrambled siRNA or si-LINC00324 2# for 48 h. Analysis of the RNA transcriptome sequencing results demonstrated that 360 genes showed differential expression (fold change > 2, *p* < 0.05), 167 of them showed increased expression in LINC00324-depleted cells, while 193 of them were downregulated (Supplementary Table1 & Fig. 6a). To verify the accuracy of the RNA transcriptome sequencing results, some functionally relevant target genes were selected and then tested by qRT-PCR assays in BGC823 and SGC7901 cells. The results illustrated that FAM83B, FOXN1, UTP20, CDH1, PRKDC, MDM2, and RB1 showed decreased expression after knockdown of LINC00324, but the expression level of SERPINB3, INHBA, CSF3, and ARNT2 were increased after LINC00324 knockdown (Fig. 6b & Supplementary FigureS1b). These data approximately matched the RNA transcriptome sequencing results and indicated that the dysregulated genes might be the potential downstream mediators of LINC00324. Since our study mainly focused on the tumorigenesis regulated by lncRNAs, which is characterized by rapid proliferation, the following experiments principally centered on this aspect. Of all the downregulated genes related with proliferation, FOXN1, FAM83B, and UTP20 showed the first three highest downregulation level (Supplementary Table2). Then we noticed FAM83B, this gene showed a high fold change of downregulation not only in LINC00324-depleted SGC7901 and BGC823 cells, but also in HuR-depleted ones (Fig. 6b). To explore whether LINC00324 could directly regulate FAM83B after binding with HuR, we used bioinformatics methods to predict the binding abundance between HuR and FAM83B. The results showed a high binding abundance between HuR and FAM83B (Fig. 6c). Then, the RIP assays validated the combination between HuR and FAM83B (Fig. 6d). Based on these data, we speculated that FAM83B might be an important downstream gene of LINC00324, and LINC00324 could enhance its stability by binding to HuR.

To further investigate whether the LINC00324-HuR complex could regulate the stability of FAM83B, we respectively transfected LINC00324 and HuR siRNAs into both BGC823 and SGC7901 cells and treated these GC cells with Actinomycin D(actD). Cells were harvested every 3 h for obtaining RNA and qRT-PCR detection. The results demonstrated that the mRNA level of FAM83B was significantly reduced in half-life after knockdown of LINC00324 or HuR compared to the control group (Fig. 6e & Supplementary FigureS1c). In summary, these results suggested that LINC00324 could enhance the stability of FAM83B through binding to HuR.

### FAM83B is involved in the LINC00324-induced GC cell proliferation

**Fig. 7:**
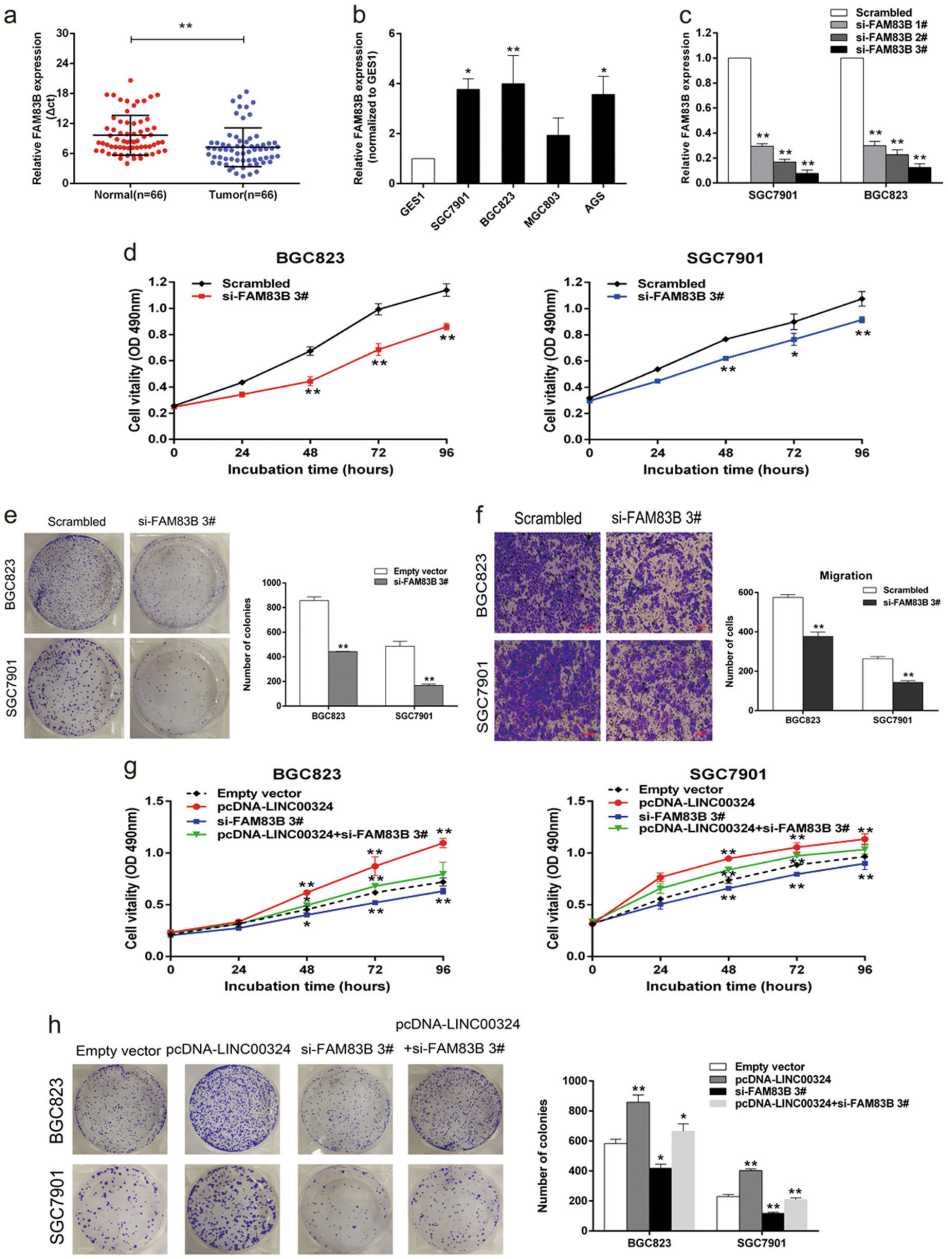
Increased expression of FAM83B promotes GC cell proliferation and is involved in the oncogene function of LINC00324. **a** Relative expression of FAM83B in 66 paired human GC tissues and their corresponding adjacent non-tumor tissues were analyzed by qRT-PCR and normalized against GAPDH expression. The data are presented as the delta CT value. **b** qRT-PCR analysis of FAM83B expression in normal gastric epithelium cell line (GES1) and GC cells. **c** qRT-PCR analysis of FAM83B expression in BGC823 and SGC7901 cells transfected with control (scrambled), si-FAM83B 1#, si-FAM83B 2# and si-FAM83B 3#. **d**, **e** MTT and colony-formation assays were performed to determine the viability of BGC823 and SGC7901 cells transfected with si-FAM83B 3# or control (scrambled). Experiments were performed in triplicate. **f** Migration ability was investigated by transwell assays in BGC823 and SGC7901 cells transfected with si-FAM83B 3# or control (scrambled). **g**, **h** MTT and colony-formation assays were used to determine the cell viability of si-FAM83B 3# and pcDNA-LINC00324 co-transfected BGC823 and SGC7901 cells. Experiments were performed in triplicate. Values are shown as the mean ± standard errors of the mean from three independent experiments. *P < 0.05, **P < 0.01

In order to further investigate the relationship between FAM83B and LINC00324 expression in GC cells, we examined the expression level of FAM83B in 66 pairs of GC tissues and their corresponding normal tissues as well as several GC cell lines by qRT-PCR. As shown in Fig. 7a, b, the expression level of FAM83B was remarkably increased in GC tissues and cells compared with normal ones. To validate the effect of FAM83B on the proliferation of GC cells, FAM83B expression was knocked down in SGC7901 and BGC823 cells (Fig. 7c). MTT and colony-formation assays revealed that knockdown of FAM83B could significantly inhibit the proliferation of GC cells (Fig. 7d, e). Transwell assays were also conducted, the result showed that knockdown of FAM83B could inhibit the migration of GC cells (Fig. 7f). These findings pointed out that FAM83B promoted the proliferation and migration of GC cells.

We also performed rescue assays to validate whether FAM83B participated in the LINC00324-induced GC cell proliferation. SGC7901 and BGC823 cells were co-transfected with pcDNA-LINC00324 and FAM83B siRNAs. The results of MTT and colony-formation assays indicated that co-transfection could partially rescue the promotion of proliferation in SGC7901 and BGC823 cells caused by the overexpression of LINC00324 (Fig. 7g, h). These evidence indicated that LINC00324 could promote the proliferation of GC cells partly through stabilizing the expression of FAM83B.

## Discussion

In recent years, more and more studies have revealed the vital roles of lncRNAs in the development and progression of numerous human diseases, including cancers^31^. Our previous study identified lncRNA HOXA-AS2 can promote GC cell proliferation via epigenetically silencing P21/PLK3/DDIT3 transcription by binding with EZH2^32^. The pseudogene derived lncRNA DUXAP8 can epigenetically silence PLEKHO1 expression by binding with PRC2, thereby promoting cell proliferation and migration in GC^33^. In addition, LINC01234 can function as a ceRNA for miR-204-5p, leading to the derepression of its endogenous target core-binding factor β (CBFB) in GC^17^. These findings suggest that the ectopic expression of lncRNAs may play an important regulatory role in the progression of GC. Moreover, lncRNAs are generally involved in cell proliferation, apoptosis, invasion, and metastasis of GC cells, and they can regulate the expression of specific genes at the transcriptional and post-transcriptional level^34^. Therefore, finding out more lncRNAs associated with GC and studying their molecular mechanisms in GC more systematically will become an important part of early diagnosis and treatment of GC, and is also of great significance for the prognosis of GC.

In this study, we focused on the lncRNA LINC00324, the function and potential mechanisms of which in GC had not been explored before. QRT-PCR was used to estimate the expression levels of LINC00324 in 66 paired GC tissues and adjacent normal tissues. The results showed that LINC00324 expression was significantly higher in GC tissues and the upregulation of LINC00324 was closely related to advanced TNM stage, larger tumor size, lymphatic metastasis, and poor prognosis of GC patients, suggesting that LINC00324 may become a molecular target for the diagnosis and prognosis of GC. Gain or loss of function assays identified that LINC00324 promoted cell proliferation and inhibited cell apoptosis, and the overexpression of LINC00324 improved the migration and invasion of GC cells. These findings indicated that LINC00324 was associated with the tumorigenesis and progression of GC.

LncRNAs can play regulatory roles by binding with different types of proteins in cancer cells. For instance, the lncRNA TINCR could combine with STAU1 (staufen1) protein, and the TINCR/STAU1 complexes directly affected the stability and expression of KLF2 mRNA. Subsequently, the downregulation of KLF2 decreased CDKN2B/P15 and CDKN1A/P21 transcription, which contributing to the cell proliferation, migration, invasion, and tumorigenicity of GC^35^. In addition, lncRNAs can also act as molecular scaffolds to regulate the expression of downstream target genes by combining with different proteins^36–38^. Since the human RBP HuR can enhance the stability of mRNA and thus induced lncRNAs expression, we then detected the possible relationship between LINC00324 and HuR using Bioinformatics. The analysis showed that LINC00324 and HuR highly integrated, and the result was verified by the RIP assay. In summary, LINC00324 can increase the stability of target mRNAs by binding with HuR, thereby promoting the proliferation of GC cells.

In order to further investigate the regulatory mechanisms of LINC00324 in GC, the RNA transcriptome sequencing was used to screen out differentially expressed genes in the cells transfected with scrambled and si-LINC00324, respectively. The results demonstrated that 360 genes showed differential expression (fold change > 2, *p* < 0.05). The results of Enrichment analysis showed that these genes were associated with many biological processes such as cell proliferation, cell adhesion, cell metastasis, and apoptosis. QRT-PCR analysis further screened out that FAM83B may be the target gene of LINC00324.

FAM83B has been identified as an oncogene that can promote the transformation of immortalized HMECs by the VBIM strategy. FAM83B is also a key intermediary in EGFR/RAS/MAPK and PI3K/AKT/mTOR signaling, and associated with cell proliferation, AIG, and tumorigenicity^27, 28^.

In this study, we treated GC cells that were transfected with si-LINC00324 and si-HuR, respectively, with actD. The results suggested that the stability of FAM83B can be enhanced by LINC00324 through binding to HuR. Furthermore, we found that the inhibition of FAM83B significantly attenuated cell growth and migration in GC cells, suggesting that FAM83B may play an oncogenic role in GC. Importantly, reduced FAM83B expression partially reversed the promotion of cell growth in GC induced by the overexpression of LINC00324. In summary, the “LINC00324-HuR-FAM83B” axis may play a crucial role in the proliferation and migration of GC. Taken together, we first revealed that LINC00324 expression was upregulated in GC tissues and cells and its upregulation may be a new biomarker for diagnosis and prognosis for patients with GC. Inhibition of LINC00324 can decrease cell proliferation and induce cell apoptosis. The oncogenic function of LINC00324 may achieve partially through stabilizing the expression of FAM83B following binding with HuR. However, further investigation should be conducted to detect other possible mechanisms of LINC00324. Nevertheless, our finding enriches the molecular mechanisms how lncRNAs regulate GC carcinogenesis and provide the experimental and theoretical evidence for the clinical diagnosis and treatment of GC.

## Materials and methods

### Tissue samples and clinical data collection

A total of 66 paired GC and corresponding normal adjacent tissues were obtained from patients who had diagnosed with GC and undergone surgery at the First Affiliated Hospital of Nanjing Medical University. None of these patients were given local or systemic treatment before surgery. All cases were verified as GC according to histopathological evaluation. The clinicopathological characteristics of GC patients are summarized in Table 1. All tissues were immediately frozen in liquid nitrogen and stored at −80 °C until needed. Our study was approved by Research Ethics Committee of Nanjing Medical University and received informed consent from all patients.

### Cell culture

Four human GC cell lines (SGC7901, BGC823, MGC803, AGS) and the normal human gastric epithelial cell line (GES-1) were purchased from the Institute of Biochemistry and Cell Biology of the Chinese Academy of Sciences (Shanghai, China). SGC7901 cells were cultured in Dulbecco’s Modifed Eagle Medium (DMEM; GIBCO-BRL) and BGC823 cells were cultured in RPMI-1640 medium (GIBCO-BRL). Both DMEM and RPMI-1640 were supplemented with 10% fetal bovine serum (FBS; ScienCell) and antibiotics (100 U/ml penicillin and 100 mg/ml streptomycin) (Invitrogen, Carlsbad, CA, USA). All cells were cultured in humidifed air at 37 °C with 5% CO2. Fresh medium was changed every 2–3 days and cells were passaged when cell confluence reached 80%−90%.

### RNA isolation and qRT-PCR analyses

Total RNA was extracted from tissues or cultured cells using TRIZOL reagent (Invitrogen). RNA (1 μg) was reverse transcribed to cDNA in a final volume of 20 μl using the PrimeScript RT Reagent Kit (Takara, Dalian, China). Real-time PCR analyses were performed using SYBR Premix Ex Taq (Takara). The results of qRT-PCR were normalized to the expression of glyceraldehyde 3-phosphate dehydrogenase (GAPDH) and data were analyzed based on the comparative cycle threshold (CT) (2^-ΔΔCT^) method. The specific primers are listed in Supplementary Table 3.

### RNA interference

SGC7901 and BGC823 cells were transfected with specific siRNAs using Lipofectamine 2000 (Invitrogen, USA). Three individual LINC00324 siRNAs (si-LINC00324 1#, 2#, 3#) and scrambled negative control siRNA (si-NC) were purchased from Invitrogen. HUR siRNA and FAM83B siRNA were synthesized by Genepharma(Shanghai, China). The nucleotide sequences of LINC00324 siRNAs, HUR siRNA, and FAM83B siRNAs are listed in Supplementary Table 3. Cells were harvested for qRT-PCR or western blot analysis 48 h after transfection.

### Plasmid generation

SGC7901 and BGC823 cells were transfected with plasmid vector using XtremeGENE HP DNA transfection reagent (Roche, Basel, Switzerland) according to the manufacturer’s instructions. The full-length complementary DNA of LINC00324 was synthesized and subcloned into the pcDNA3.1 (+) vector by Generay (Shanghai, China). Cells were harvested for qRT-PCR or western blot analysis 48 h after transfection.

### Cell proliferation assays

Cell viability was detected using Cell Proliferation Reagent Kit I (MTT; Roche Applied Science). SGC7901 and BGC823 cells transfected with si-LINC00324 or pcDNA-LINC00324 were cultured in 96-well plates (3000 cells/well). Cell viability was monitored every 24 h following the manufacture’s protocol. All experiments were performed in triplicate. For the colony-formation assay, a certain number of transfected cells were cultured in each well of 6-well plates and maintained in appropriate medium containing 10% FBS for 14 days, during this period, the medium was replaced every four days. After 14 days, the cells were fixed with methanol and stained with 0.1% crystal violet (Sigma-Aldrich). Visible colonies were then counted. Wells were performed in triplicate for each treatment group, and experiments were independently repeated three times.

### Cell migration and invasion assays

For the migration assays, SGC7901 and BGC823 cells transfected with si-LINC00324 or pcDNA-LINC00324 were cultured in 24-well plates with an 8-mm pore size polycarbonate membrane (Corning Incorporated). For the invasion assays, cells in serum-free medium were placed into the upper chamber of an insert coated with Matrigel (Sigma-Aldrich). Medium containing 10% FBS was added to the lower chamber. After 24 h, the cells remaining on the upper chamber were wiped with cotton swabs, while cells on the lower membrane surface were fixed with methanol and stained with 0.1% crystal violet after 24 h incubation. Five fields of view were randomly selected in each well for counting.

### Ethynyldeoxyuridine (EdU) analysis

The EdU labeling/detection kit (Ribobio, Guangzhou, China) was used to detect cell proliferation following the manufacturer’s protocol. Cells were cultured in 96-well plates at 5 × 10^3^ cells per well. Then 50 μM EdU labeling medium was added to cells 48 h after transfection, and they were incubated for 2 h at 37 °C under 5% CO2. Next, the cultured cells were treated with 4% paraformaldehyde (pH 7.4) for 30 min and then 0.5% Triton X-100 for 20 min at room temperature. Then the samples were stained with anti-EdU working solution and subsequently incubated with 100 μl Hoechst 33342 (5 μg/ml). The percentage of EdU-positive cells was measured under fluorescent microscopy. Five fields of view were randomly selected in each well for counting the percentage of EdU-positive cells.

### Flow cytometric analysis

SGC7901 and BGC823 cells transfected with si-LINC00324 or scrambled were harvested 48 h after transfection by trypsinisation. After double staining with FITC-Annexin V and propidium iodide (PI) by using the FITC Annexin V Apoptosis Detection Kit (BD Biosciences) following the manufacturer’s protocol, the cells were analyzed by flow cytometry (FACScan®; BD Biosciences) with CellQuest software (BD Biosciences). The cells were classified into viable, dead, early apoptotic, and apoptotic cells, and then the ratio of early apoptotic cells was compared with the control for each experiment.

### Western blot assay and antibodies

Cells protein lysates separated by 10% sodium dodecyl sulphate polyacrylamide gel electrophoresis (SDS-PAGE) were transferred to 0.22 μm NC membranes (Sigma) and incubated with specific antibodies. The ECL chromogenic substrate was was quantified by densitometry (Quantity One software; Bio-Rad). A GAPDH antibody was used as a control, and Caspase 3, Caspase 7, Caspase 9, PARP, Cleaved Caspase 3, Cleaved Caspase 7, Cleaved Caspase 9, Cleaved PARP (1:1000) were purchased from Cell Signaling Technology, Inc (CST).

### Subcellular fractionation location

The separation of nuclear and cytosolic fractions was performed using the PARIS Kit (Life Technologies, Carlsbad, CA) according to the manufacturer’s instructions.

### RNA immunoprecipitation (RIP) assay

RIP was performed using the EZ-Magna RIP kit (Millipore, Billerica, MA) following the manufacturer’s protocol. SGC7901 and BGC823 cells at 80–90% confluency were scraped off and then lysed in complete RIP lysis buffer. A total of 100 μl of whole cell extract was incubated with RIP buffer containing magnetic beads conjugated with antibodies against HuR or control IgG (Millipore) for 6 h at 4 °C. The beads were then washed with washing buffer, the complexes were incubated with 0.1% SDS/0.5 mg/ml Proteinase K (30 min at 55 °C) to remove proteins. The RNA concentration was measured by NanoDrop spectrophotometer (Thermo Scientific), and the RNA quality was assessed using a bioanalyser (Agilent, Santa Clara, CA). Finally, immunoprecipitated RNA was purified and analyzed by qRT-PCR.

### Tumor formation assay in a nude mouse model

Male BALB/c-nude mice (4-week-old) were maintained under specific pathogen-free (SPF) conditions and manipulated according to protocols approved by the Shanghai Medical Experimental Animal Care Commission. SGC7901 cells were stably transfected with sh-LINC00324 or empty vector and harvested from 6-well cell culture plates, washed with phosphate-buffered saline (PBS), and re-suspended at a concentration of 1 × 10^8^ cells/ml. A total of 100 μl of suspended cells was subcutaneously injected into a single side of the armpit of each mouse. Tumor growth was detected every five days, and tumor volumes were calculated using the equation *V* = 0.5 × D × d^2^ (*V* means volume; *D* means longitudinal diameter; *d* means latitudinal diameter). At 15 days post-injection, the mice were euthanized, and the subcutaneous growth of each tumor was examined. This study was performed strictly in accordance with the recommendations in the Guide for the Care and Use of Laboratory Animals of the National Institutes of Health. The protocol was approved by the Animal Ethical and Welfare Committee of Nanjing Medical University.

### Immunohistochemical (IHC) analysis

The primary tumors were immunostained for Ki-67 as previously described.

### Statistical analysis

All statistical analyses were performed using SPSS 17.0 software (IBM, SPSS, USA). The signifcance of differences between groups was estimated by a paired, two-tailed Student’s *t*-test, χ2 test, or Wilcoxon test, as appropriate. DFS and OS rates were calculated by the Kaplan–Meier method with the log-rank test applied for comparison. *P*-values less than 0.05 were recognized as significant.

## Electronic supplementary material

Supplementary Table 1

Supplementary Table 2

Supplementary Table 3

Figure S1

supplementary figure legends
