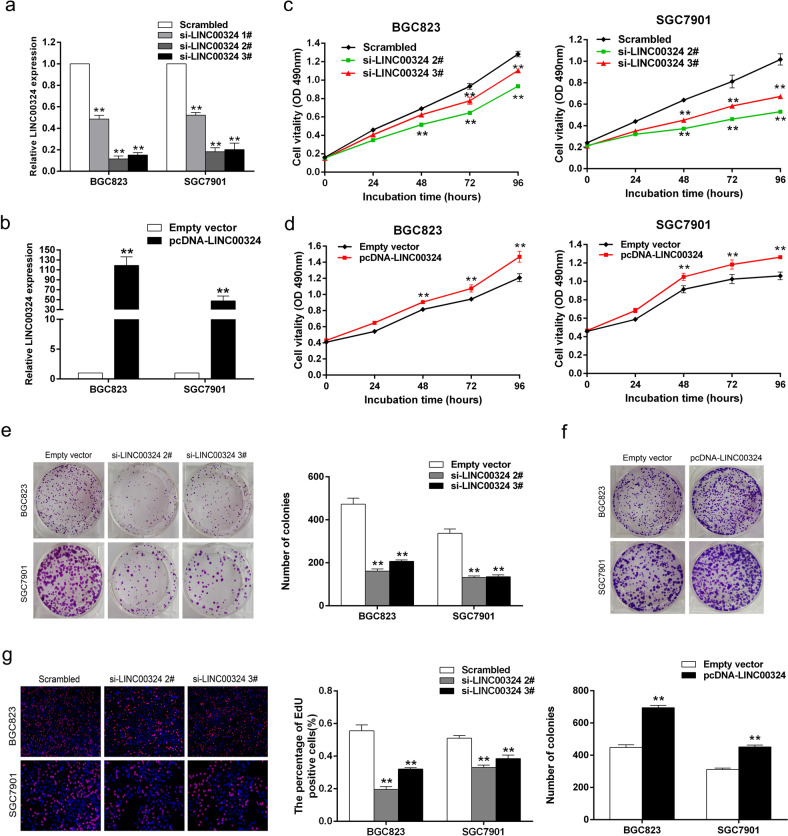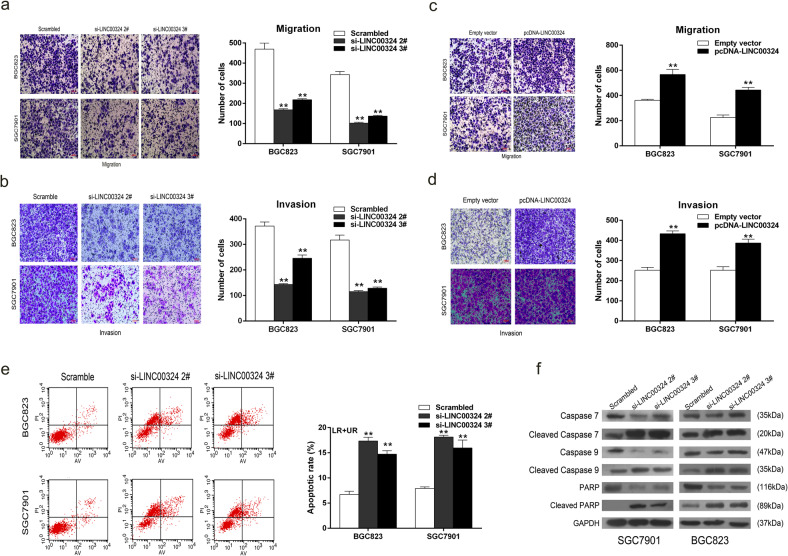# Correction: Long intergenic non-coding RNA 00324 promotes gastric cancer cell proliferation via binding with HuR and stabilizing FAM83B expression

**DOI:** 10.1038/s41419-022-05232-7

**Published:** 2022-09-09

**Authors:** Zigui Zou, Tianshi Ma, Xuezhi He, Jinxing Zhou, Hongwei Ma, Min Xie, Yanhua Liu, Die Lu, Shihao Di, Zhihong Zhang

**Affiliations:** 1 Department of Pathology, The First Affiliated Hospital of Nanjing Medical University, Nanjing, China; 2 Department of Anatomy, Histology and Embryology, Research Centre for Bone and Stem Cells, Nanjing Medical University, Nanjing, China; 3 Department of Pathology, Zhenjiang First people’s hospital, Zhenjiang, China; 4 Central Laboratory, Suzhou Municipal Hospital, Nanjing Medical University Affiliated Suzhou Hospital, Suzhou, China; 5 Department of Oncology, Affiliated Xuzhou Central Hospital, Xuzhou Medical College, Xuzhou, China

Correction to: *Cell Death and Disease* 10.1038/s41419-018-0758-8, published online 18 June 2018

The original version of this article contained errors in figures 2 and 3. The author apologize for the errors. The corrected figures can be found below.