# Supplementary material for: Long intergenic non-coding RNA 00324 promotes gastric cancer cell proliferation via binding with HuR and stabilizing FAM83B expression
Source: Cell Death Dis. 2018 Jun 18;9(7):717. doi: 10.1038/s41419-018-0758-8 (PMC6006375; doi:10.1038/s41419-018-0758-8)
Supplement: Supplementary file 1 — Supplementary Table 1 [file 41419_2018_758_MOESM1_ESM.docx]

| mRNAs | Regulation | Ratio | mRNAs | Regulation | Ratio |
| --- | --- | --- | --- | --- | --- |
| CSF3 | Up | 7.0319 | CDH1 | Down | 3.74042 |
| CCL3 | Up | 5.943378 | FOXN1 | Down | 3.30783 |
| MMP1 | Up | 5.912395 | FAM83B | Down | 2.62568 |
| INHBA | Up | 5.412814 | SYTL5 | Down | 2.62232 |
| KRT75 | Up | 5.17079 | IFIT2 | Down | 2.61023 |
| SERPINB7 | Up | 5.069512 | SULT2B1 | Down | 2.56814 |
| ZP4 | Up | 4.468616 | CCDC121 | Down | 2.54304 |
| GPRASP2 | Up | 4.413349 | SAPCD1 | Down | 2.54053 |
| CXCL8 | Up | 4.352779 | LY6D | Down | 2.53632 |
| CCL3L3 | Up | 4.323652 | RFX7 | Down | 2.51004 |
| TRPA1 | Up | 4.302573 | IFIT1 | Down | 2.50839 |
| UGT1A3 | Up | 4.269532 | PRKDC | Down | 1.96211 |
| CCL17 | Up | 4.176477 | PLEKHA2 | Down | 1.94784 |
| SERPINB3 | Up | 3.693339 | USF3 | Down | 1.8656 |
| ARNT2 | Up | 2.027365 | MDM2 | Down | 1.85852 |

| **Supplementary Table1: Part of differentially expressed mRNAs in 7901 cells transfected with si-LINC00324 determined by RNA transcriptome sequencing** |
| --- |
|  |
|  |
|  |
|  |
|  |
|  |
|  |
|  |
|  |
