# Supplementary material for: Long intergenic non-coding RNA 00324 promotes gastric cancer cell proliferation via binding with HuR and stabilizing FAM83B expression
Source: Cell Death Dis. 2018 Jun 18;9(7):717. doi: 10.1038/s41419-018-0758-8 (PMC6006375; doi:10.1038/s41419-018-0758-8)
Supplement: Supplementary file 2 — Supplementary Table 2 [file 41419_2018_758_MOESM2_ESM.docx]

| **mRNAs** | **Regulation** | **Ratio** | **mRNAs** | **Regulation** | **Ratio** |
| --- | --- | --- | --- | --- | --- |
| FOXN1 | Down | 3.30783 | PTGS1 | Down | 2.20991 |
| FAM83B | Down | 2.62568 | DOCK8 | Down | 2.20186 |
| UTP20 | Down | 2.58276 | FGF18 | Down | 2.18147 |
| FGF21 | Down | 2.44928 | CXCL10 | Down | 2.04227 |
| IFNL1 | Down | 2.44516 | CRIP2 | Down | 2.01764 |
| INSL4 | Down | 2.43939 | ACE | Down | 2.01611 |
| FGFBP1 | Down | 2.35111 | KMT2A | Down | 2.01258 |
| INPP5D | Down | 2.22750 | PRKDC | Down | 1.96211 |

**Supplementary Table2: Part of downregulated mRNAs related with cell proliferation in 7901 cells transfected with si-LINC00324 determined by RNA transcriptome sequencing**
