# Supplementary figures and images for: Long intergenic non-coding RNA 00324 promotes gastric cancer cell proliferation via binding with HuR and stabilizing FAM83B expression
Source: Cell Death Dis. 2018 Jun 18;9(7):717. doi: 10.1038/s41419-018-0758-8 (PMC6006375; doi:10.1038/s41419-018-0758-8)

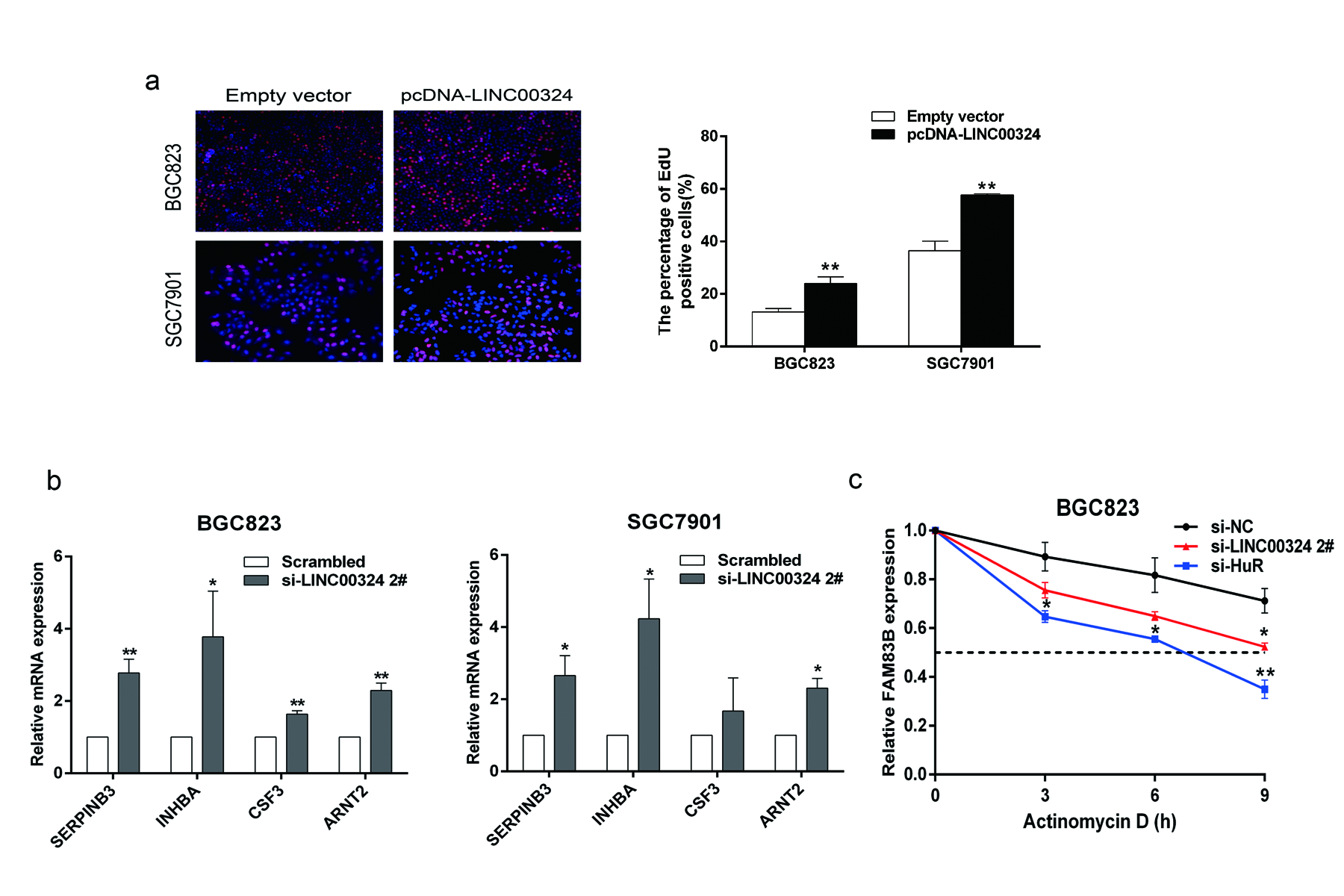

Supplement: Supplementary file 4 — Figure S1 [file 41419_2018_758_MOESM4_ESM.tif]
