# Supplementary material for: Long intergenic non-coding RNA 00324 promotes gastric cancer cell proliferation via binding with HuR and stabilizing FAM83B expression
Source: Cell Death Dis. 2018 Jun 18;9(7):717. doi: 10.1038/s41419-018-0758-8 (PMC6006375; doi:10.1038/s41419-018-0758-8)
Supplement: Supplementary file 5 — supplementary figure legends [file 41419_2018_758_MOESM5_ESM.docx]

**Supplementary information**

**Supplementary Table 1:** Part of differentially expressed mRNAs in 7901 cells transfected
with si-LINC00324 determined by RNA transcriptome sequencing. (.docx 17kb)

**Supplementary Table 2:** Part of downregulated mRNAs related with cell proliferation in 7901 cells transfected with si-LINC00324 determined by RNA transcriptome sequencing. (.docx 13.8kb)

**Supplementary Table 3:** Sequences of primers, siRNAs and shRNA, information about antibodies. (.xlsx 11.9kb)

**Supplementary Figure S1:** Effects of LINC00324 on proliferation using EdU assays,validation of RNA transcriptome sequencing and effects of LINC00324 on the stability of FAM83B through binding to HuR . (a) EdU staining assays were used to determine the proliferation of BGC823 and SGC7901 cells transfected with pcDNA-LINC00324. EdU positive cells were counted and captured. Values are shown as the mean ± standard errors of the mean based on three independent experiments. *P < 0.05, **P < 0.01. (b) QRT-PCR analysis was used to validate the changes of several mRNAs involved in cell proliferation and cell migration upon LINC00324 or HuR depletion. (c) RNA stability assays were performed using Actinomycin D to disrupt RNA synthesis in BGC823 cells, and the degradation rates of the FAM83B mRNAs were measured every 3 h. Values are shown as the mean± standard errors of the mean from three independent experiments. *P < 0.05, **P < 0.01.
